# Not all *NTRK* fusions in mesenchymal neoplasia are driver events: implications on classification and targeted therapy

**DOI:** 10.1002/2056-4538.70111

**Published:** 2026-08-02

**Authors:** Mohamed A Yakoub, Purvil Sukhadia, Carla Saoud, Meera Hameed, Cristina R Antonescu

**Affiliations:** ^1^ Department of Pathology and Laboratory Medicine Memorial Sloan Kettering Cancer Center New York NY USA

**Keywords:** *NTRK*, fusion, driver, sarcoma, *NTRK* fusions, oncogenic driver, passenger fusion, targeted therapy, TRK inhibitors, *MDM2* amplification

## Abstract

*NTRK* fusions drive the pathogenesis of a distinctive group of mesenchymal neoplasms with significant impact on classification and targeted therapy. However, unexpected *NTRK* fusions have been reported in other sarcoma entities, raising uncertainty over their specificity and clinical management. Herein, we investigate the incidence and structural variants of *NTRK* fusions among a large clinicopathologic and molecular sarcoma cohort. The goal was to distinguish primary driver *NTRK* fusions from potential passenger events and correlate with sarcoma histotypes. *NTRK1‐3* fusions were queried across a large spectrum of sarcomas, profiled by targeted DNA and/or RNAseq. Fusions were classified as oncogenic when *NTRK* was the 3′ partner, in‐frame, retained kinase domain (KD), and/or RNAseq confirmation; fusions of uncertain significance (FUS) when out‐of‐frame, lacking full KD, and/or RNAseq negative. We identified 48 cases with *NTRK* fusions, detected either by pathologist‐initiated RNAseq for diagnosis (*n* = 27) or by clinician‐initiated DNAseq for therapeutic target discovery (*n* = 21). For the latter subset, reflex RNAseq was activated for confirmation. Integrated review confirmed 33 (69%) oncogenic fusions. Remaining were FUS, apart from one indeterminate. In all except three cases, oncogenic fusions occurred in canonical *NTRK*‐driven histotypes. In contrast, *NTRK* FUS were detected in various pathologic entities, including well‐differentiated/dedifferentiated liposarcoma (*n* = 9, 60%), two osteosarcoma, and single cases of other subtypes. *NTRK1* fusions were the most common in both oncogenic and FUS groups (61%, 67%). *CDKN2A/B* deletions were observed mostly in oncogenic *NTRK1* fusions (71%), while *MDM2/CDK4* amplifications in the *NTRK1* FUS. The FUS group demonstrated lower *NTRK* mRNA expression, with a mean of −4.60, *p* < 0.001. Pan‐TRK immunohistochemistry was positive in cases with oncogenic *NTRK* fusions, and negative in FUS cases. Only two‐thirds of *NTRK* fusions detected were functional drivers, mostly from pathologist‐driven testing of suggestive histotypes. In contrast, genomic profiling in complex sarcomas often yields passenger FUS, lacking functional impact.

## Introduction

The neurotrophic receptor tyrosine kinase (*NTRK*) gene family, which includes *NTRK1*, *NTRK2*, and *NTRK3*, encodes the tropomyosin receptor kinase (TRK) proteins [1]. *NTRK* fusions have been shown to be oncogenic drivers, whereby the fusion of the 3′ region of an *NTRK* gene, containing the intact kinase domain, with the 5′ end of a partner gene leads to a chimeric protein. The partner gene provides a dimerization domain that causes ligand‐independent, constitutive activation of the TRK kinase, leading to unchecked activation of downstream signaling pathways, such as the RAS/MAPK and PI3K/AKT pathways, thereby promoting tumorigenesis [1, 2].

Beyond the classic example of infantile fibrosarcoma, an emerging family of *NTRK*‐rearranged spindle cell neoplasms has been recognized, including the recently described lipofibromatosis‐like neural tumor, which is often characterized by *NTRK1* fusions [3]. These tumors frequently display a recurring histologic theme of monomorphic spindle cell morphology with variable stromal and perivascular hyalinization and frequent co‐expression of S100 and CD34 proteins [4, 5]. Based on these findings, the recent World Health Organization (WHO) Classification of Soft Tissue and Bone Tumors has formally included ‘*NTRK*‐rearranged spindle cell neoplasm’ as a stand‐alone tumor category, acknowledging its clinical and pathological significance [6].

However, the increasing use of broad next‐generation sequencing (NGS) panels has shown unexpected *NTRK* fusions being detected in a variety of common sarcoma subtypes that are not historically associated with these alterations and are defined by other known driver events [7]. This raises the critical question of whether all detected *NTRK* fusions are *bona fide* oncogenic drivers or represent incidental passenger events in tumors driven by alternative mechanisms. The distinction is of paramount importance, diagnostically and prognostically; hence the aim of this study.

## Materials and methods

### Clinical and molecular cohort

After approval by the Institutional Review Board, all study subjects provided written informed consent to the use of their genomic data for research (IRB# 12‐245). We retrospectively queried our institutional molecular pathology database and cBioPortal (https://www.cbioportal.org; last accessed 12 May 2025) [8]; between 2016 and 2025, to identify all sarcoma cases that had undergone clinical molecular profiling, using targeted DNA and/or RNAseq, and were reported to have a gene fusion involving *NTRK1*, *NTRK2*, or *NTRK3*. All cases underwent a comprehensive clinicopathologic review by sarcoma pathologists to confirm the histologic diagnosis according to the criteria of the 2020 WHO Classification of Soft Tissue and Bone Tumors. Clinical data, including age, sex, and tumor location, were extracted from the electronic medical records.

### Targeted NGS

Testing of eligible cases which revealed *NTRK1‐3* used pathologist‐initiated RNAseq (anchored multiplex PCR‐based NGS known as Archer FusionPlex; Integrated DNA Technologies, Inc., Coralville, IA, USA), typically in the context of a suspected kinase‐fusion‐driven neoplasm, to confirm diagnosis [9]. In another set of cases, the fusion was detected by clinician‐initiated DNAseq for therapeutic target discovery, using the Memorial Sloan Kettering Cancer Center – Integrated Mutation Profiling of Actionable Cancer Targets (MSK‐IMPACT™), a comprehensive molecular assay that involves hybridization capture and deep sequencing of all exons and selected introns of 341–505 oncogenes and tumor‐suppressor genes, allowing the detection of point mutations, small and large insertions, deletions, and structural variants, including select gene fusions [10].

### Fusion classification and genomic analysis

All identified *NTRK* fusions were subject to a rigorous classification schema. Fusions were defined as Oncogenic (Driver) if they met the following criteria: (1) the *NTRK* gene constituted the 3′ partner of the fusion, (2) the fusion was predicted to be in‐frame, (3) the fusion resulted in the retention of the entire *NTRK* kinase domain, and (4) for fusions initially detected by DNA‐seq, the fusion transcript was confirmed by reflex RNA‐seq. Fusions that did not meet these criteria were classified as fusions of uncertain significance (FUS). This category included fusions that were out‐of‐frame, resulted in a truncated kinase domain, or were detected by DNA‐seq but not confirmed by subsequent RNA‐seq.

### Pan‐TRK immunohistochemistry

Selected *NTRK* FUS cases, and majority of Oncogenic *NTRK* fusion positive cases, underwent Pan‐TRK IHC (clone: EPR17341; Abcam, Waltham, MA, USA) as previously described [11].

### mRNA expression by RNA‐Seq

Relative RNA expression levels of *NTRK1*, *NTRK2*, and *NTRK3* for selected individual cases were collected from Archer FusionPlex data and quantitatively assessed by colorimetric analysis of heatmap visualizations. The highest expression for each *NTRK* gene within each case was mapped to a Log Base 2 scale, ranging from +6 to −6. The means of RNA expression of the corresponding *NTRK* gene were used for group comparisons.

*NTRK1/2/3* mutations, as well as co‐occurring genomic alterations, including CNAs such as *CDKN2A/B* deletions and *MDM2/CDK4* amplifications, were also analyzed in relation to the *NTRK* fusion status. Fisher's exact test was used to calculate statistical significance. Data visualization was performed using Oncoprints generated with R version 4.1.3 (R Foundation for Statistical Computing, Vienna, Austria; https://www.R-project.org; last accessed 28 March 2026).

## Results

### Identification and classification of *NTRK* fusions

We identified a total of 48 sarcoma cases with a reported *NTRK* gene fusion. The fusion detection included: 27 cases (56%) were identified by pathologist‐initiated targeted RNA sequencing, and 21 cases (44%) were identified by clinician‐initiated targeted DNA sequencing (MSK‐IMPACT). For this latter group, reflex RNA sequencing was performed for orthogonal confirmation of the fusion transcript in 20 cases.

**Figure 1:**
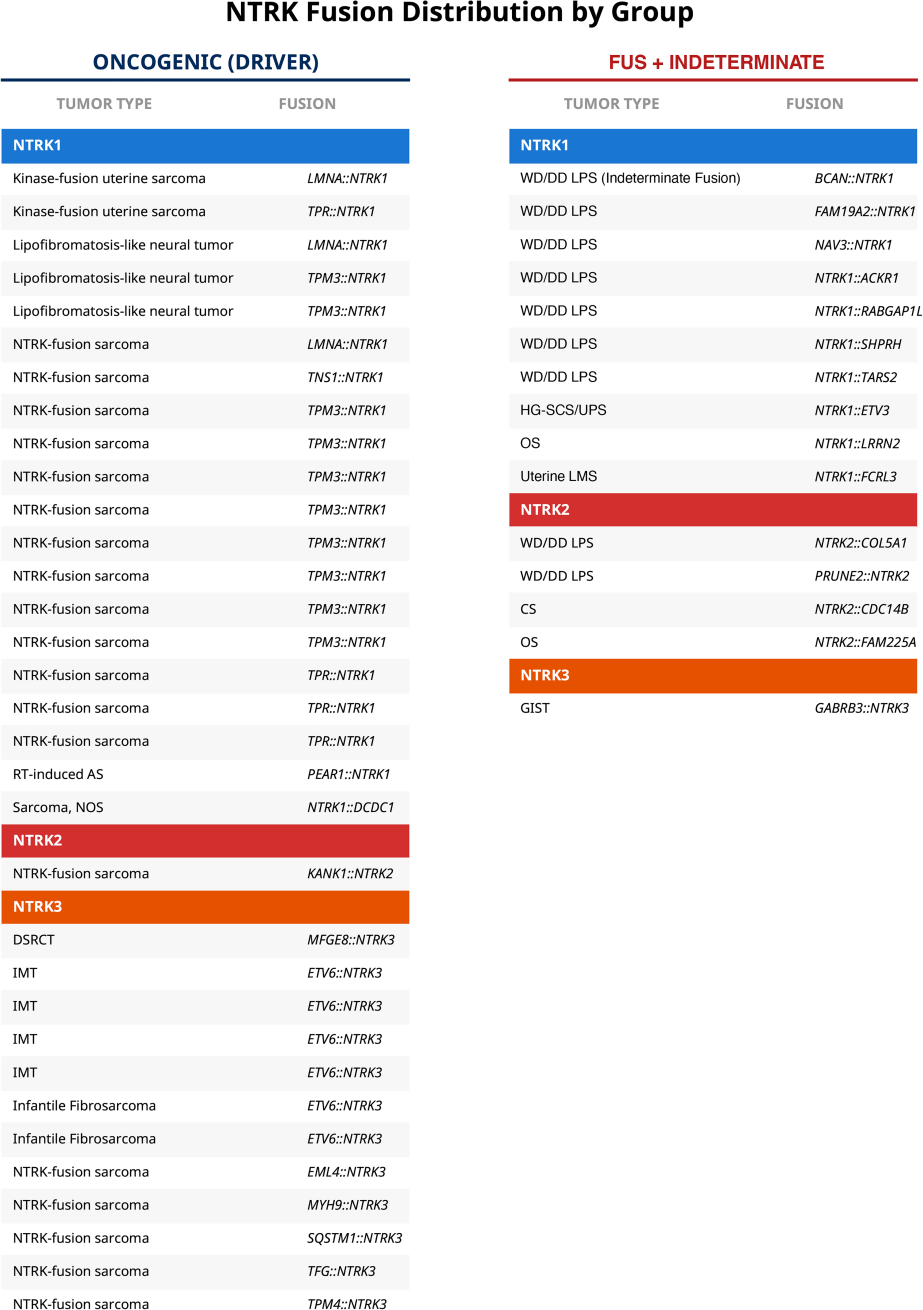
*NTRK* fusion distribution by group. *NTRK* fusion events identified in the cohort, stratified into Oncogenic (Driver) and FUS (Fusions of Uncertain Significance) groups. CS, chondrosarcoma; DSRCT, desmoplastic small round cell tumor; GIST, gastrointestinal stromal tumor; HG‐SCS/UPS, high‐grade spindle cell sarcoma/undifferentiated pleomorphic sarcoma; IMT, inflammatory myofibroblastic tumor; LMS, leiomyosarcoma; OS, osteosarcoma; WD/DD LPS, well‐differentiated/dedifferentiated liposarcoma.

Following a comprehensive integrative review of the molecular and clinicopathologic data, the 48 fusions were classified into three groups. A total of 33 fusions (69%) were classified as Oncogenic (Driver) events. The remaining cases included 14 fusions (29%) classified as FUS and one case classified as Indeterminate, as it represented a structurally oncogenic fusion at the DNA level but lacked available material for RNA sequencing confirmation. A list of all fusions in the entire cohort is demonstrated in Figure 1.

### Clinicopathologic and genomic features of oncogenic *NTRK* fusions

In this group, there were 21 females and 12 males, the median age was 34 (range: 5–71). The 33 oncogenic *NTRK* fusions were predominantly found in tumors belonging to the established spectrum of kinase‐fusion‐associated sarcomas, that is, canonical *NTRK*‐driven entity (30/33, 91%), these included kinase‐associated spindle cell tumors (*n* = 19), inflammatory myofibroblastic tumor (*n* = 4), lipofibromatosis‐like neural tumor (*n* = 3), infantile fibrosarcoma (*n* = 2), and kinase‐fusion uterine sarcoma (*n* = 2). Interestingly, additional three cases (9%), outside of this spectrum were found to harbor an oncogenic *NTRK* driver, and included one radiation‐associated angiosarcoma, one desmoplastic small round cell tumor (DSRCT), and one case of sarcoma, NOS.

The sarcoma NOS case showed an *NTRK1::DCDC1* fusion by RNA sequencing, and despite *NTRK1* being the 5′ partner gene, this fusion was deemed oncogenic, as it was found to be in‐frame, it retained the entire *NTRK1* kinase domain, and it showed increased *NTRK1* mRNA expression.

**Figure 2:**
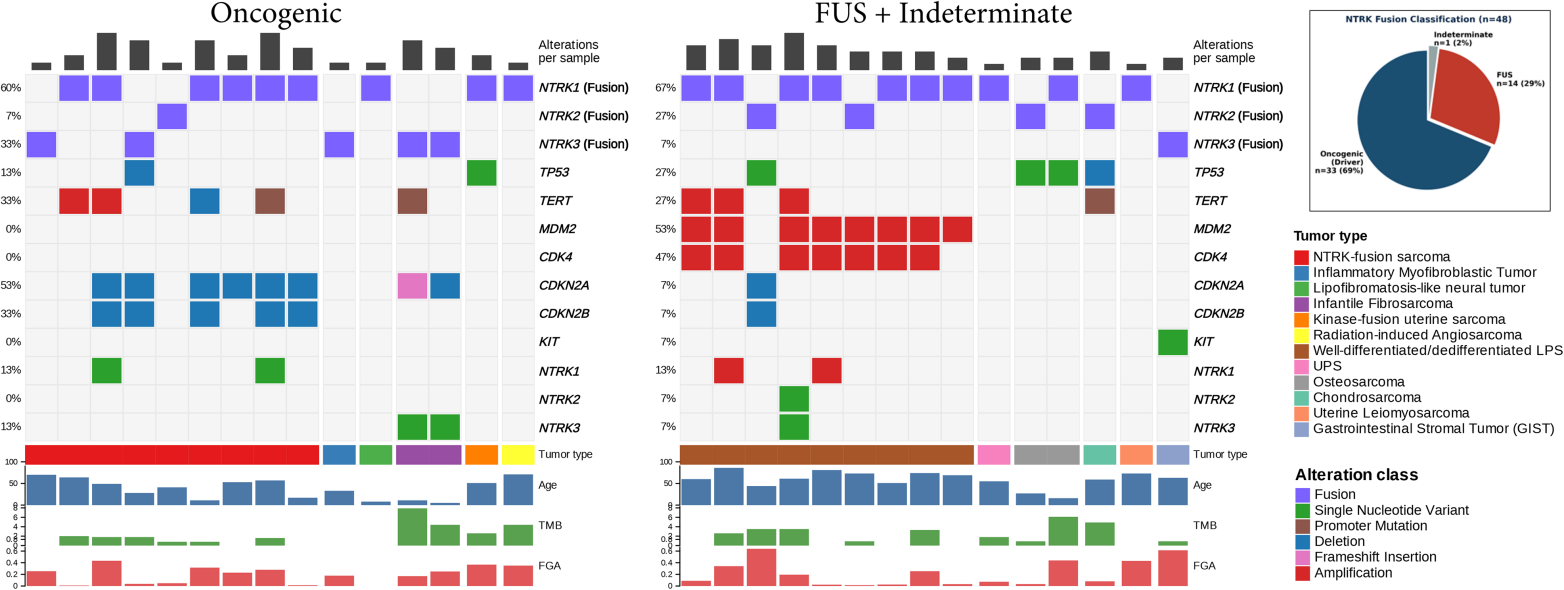
Genomic landscape of *NTRK* fusions in mesenchymal neoplasms. Oncoprint summarizing genomic alterations in the subset of oncogenic *NTRK* fusion cases that underwent DNA‐based NGS testing (15 of 33 cases), together with all FUS/indeterminate cases with available DNA‐based NGS data (*n* = 15), with tracks for gene alterations, tumor type, age, TMB, and FGA. An inset pie chart summarizes the overall *NTRK* fusion classification.

**Figure 3:**
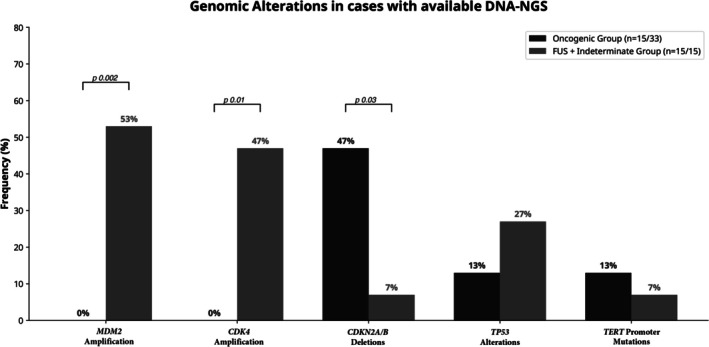
Genomic alterations in *NTRK* fusion cases. Bar plot illustrating the frequency of key genomic alterations among cases with available DNA‐based NGS data in the Oncogenic Group (15 of 33 cases) and the FUS/Indeterminate Group (15 of 15 cases). Significant differences were observed for *MDM2* amplification, *CDK4* amplification, and *CDKN2A/B* deletion.

Of the 33 cases with oncogenic *NTRK* fusions, 15 underwent DNA‐based NGS by MSK‐IMPACT (Figure 2). This subset exhibited a relatively quiet genomic background, with a mean tumor mutational burden of 1.9 mutations/Mb and a mean fraction of genome altered of 19%. Notably, five tumors had no detectable mutations, and six harbored only one to two mutations, supporting an overall low‐complexity mutational profile in this subset. *NTRK1* fusions were the most frequent, accounting for 20/33 cases (61%) in this group, followed by *NTRK3* fusions (12/33, 36%), while *NTRK2* fusion was the least prevalent, recorded in only one patient (3%). Overall co‐occurring alterations in established cancer driver genes were infrequent (Figures 2 and 3). Notably, homozygous deletions and frameshift deletions of *CDKN2A/B* were observed in 7/15 cases (47%); 5 out of these (71%) were seen in association with oncogenic *NTRK1* fusions, and 2 (29%) were seen in *NTRK3* fusions. Interestingly, five of the cases with *CDKN2A/B* deletions (71%) had high‐grade features and/or aggressive clinical behavior, with three patients developing distant metastasis, one developing local recurrence, and three patients (43%) succumbing to disease.

*TERT* alterations were the second most common, observed in 5/15 cases (33%); only 2 of these were promoter mutations. Mutations in *TP53* were rare (13%), and the tumors largely lacked other canonical sarcoma‐defining driver alterations.

Of note, four cases in the oncogenic group harbored *NTRK* single nucleotide variants (SNV) in addition to the *NTRK* fusion. Two of these were missense point mutations in exon 16 of *NTRK3*, both observed in *ETV6::NTRK3*‐positive infantile fibrosarcomas (Figure 2). In the first case, an *NTRK3* p.G623R mutation was detected in the recurrent tumor, status post 8 months of larotrectinib therapy. In the second case, the *NTRK3* p.F617I mutation also occurred in the recurrent tumor, 5 months after larotrectinib treatment. Among the nine *NTRK1*‐fusion sarcomas, two cases (22%) harbored additional *NTRK1* missense point mutations. One case showed an *NTRK1* exon 14 p.G595R mutation in a lung metastasis 10 months after entrectinib therapy, followed by acquisition of an additional *NTRK1* exon 15 p.G667C mutation in a subsequent lung metastasis. The second case harbored an *NTRK1* exon 15 p.G667C mutation in a recurrent tumor sampled 18 months after larotrectinib therapy. These findings are consistent with acquired on‐target resistance alterations involving the *NTRK1* kinase domain after TRK inhibitor treatment.

### Clinicopathologic and genomic features of *NTRK* FUS and indeterminate fusions

This group included 10 females and 5 males; the median age was 55 years (range: 16–86). In stark contrast, the 14 cases in the FUS group and the one case of indeterminate annotation occurred in a variety of sarcoma histotypes not typically associated with primary *NTRK* rearrangements. The most frequent entity in this group was well‐differentiated/dedifferentiated liposarcoma (WD/DDLPS), 9/15 (60%); of which eight harbored FUS and one was indeterminate. The remaining cases included osteosarcoma (*n* = 2), chondrosarcoma (*n* = 1), undifferentiated pleomorphic sarcoma (*n* = 1), uterine leiomyosarcoma (*n* = 1), and *KIT* exon 11 mutant gastrointestinal stromal tumor (*n* = 1).

The genomic landscape of the FUS group was significantly more complex and was defined by the presence of other well‐established primary oncogenic drivers. All liposarcoma cases showed the consistent co‐occurrence of high‐level amplification of the 12q13–15 region, including the pathognomonic drivers *MDM2* and *CDK4*, which were entirely absent from the oncogenic fusion group. Furthermore, the FUS group showed a higher frequency of *TP53* mutations (27%) and a higher fraction of genome altered (FGA) compared to the oncogenic group (23%). While *NTRK1* fusions were also the most common in this cohort (67%), *NTRK2* fusions were more common in this group compared to the oncogenic group, with a prevalence of 27% (4/15 cases) which was statistically significant (Table 1). *NTRK3* was only seen in one case. Overall, the defining feature in this group was the presence of a known, dominant, histology‐defining driver alteration.

**Table 1:** Demographics and molecular data of Oncogenic versus FUS groups

Feature	Oncogenic (*n* = 33)	FUS + Indeterminate (*n* = 15)	*p*
Median age (years)	34 (5–71)	55 (16–86)	<0.001
Sex (F/M)	21/12	10/5	–
Canonical *NTRK* entity	91% (30/33)	0% (0/15)	<0.001
WD/DD LPS	0% (0/33)	60% (9/15)	<0.001
*NTRK1* fusion	61%	67%	0.7
*NTRK2* fusion	3%	27%	0.04
*NTRK3* fusion	36%	7%	0.06
*MDM2/CDK4* Amp	0% (0/15)	53% (8/15)	<0.01
*CDKN2A/B* deletion	47% (7/15)	7% (1/15)	0.03
*TP53* mutation	13% (2/15)	27% (4/15)	0.5
Mean TMB (mut/Mb)	1.9	1.9	0.9
Mean FGA (%)	19%	23%	0.7

Statistics related to genomic alterations (*MDM2*, *CDK4*, *CDKN2A/B*, and *TP53*) and molecular metrics (FGA and TMB) were calculated based on cases with available DNA‐NGS, including 15 cases in the Oncogenic group and all 15 cases in the FUS/Indeterminate group.

FGA, fraction of genome altered; FUS, fusion of uncertain significance; TMB, tumor mutational burden.

Of note, two WD/DDLPS with *NTRK1* fusions also showed *NTRK1* amplification, suggesting that the nonfunctional *NTRK1* rearrangement could be part of the gene amplification event. Additionally, one WD/DDLPS with *NTRK1* fusion had both *NTRK1* and *NTRK2* point mutations.

### *NTRK* mRNA expression by RNA‐Seq and Pan‐TRK immunohistochemistry

**Figure 4:**
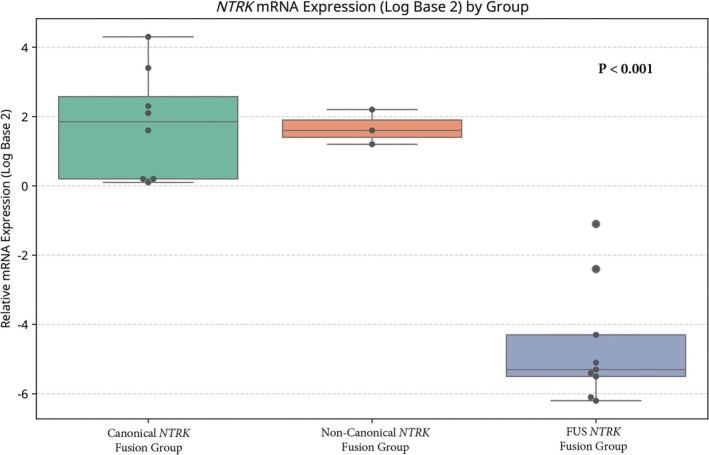
Relative *NTRK* mRNA expression by group. Boxplot demonstrating the distribution of *NTRK* mRNA expression levels across canonical, noncanonical, and FUS (fusions of uncertain significance) groups. Each box represents the interquartile range (IQR), with the median indicated by the horizontal line. Individual data points are shown as dots. A significant difference in expression (*p* < 0.001) was observed between the groups, with the FUS group exhibiting markedly lower expression compared to both canonical and noncanonical groups.

Relative mRNA expression levels of *NTRK* genes (Log Base 2) were quantitatively assessed across selected cases with available RNA expression data; canonical *NTRK* fusion cases (*n* = 8), noncanonical *NTRK* fusion cases (*n* = 3), and FUS (*n* = 9). Descriptive statistics revealed distinct expression profiles among the groups (Table 2). The canonical group exhibited a mean expression of 1.78 (SD ± 1.57), while the noncanonical group showed a mean of 1.67 (SD ± 0.50). In stark contrast, the FUS group demonstrated significantly lower expression, with a mean of −4.60 (SD ± 1.74), *p* < 0.001 (post hoc analysis using Tukey HSD). No significant difference was observed between the canonical and noncanonical *NTRK* fusion groups (mean difference = −0.11, *p* = 0.994) (Figure 4).

**Table 2:** Descriptive statistics of *NTRK* mRNA expression (Log Base 2) by group (selected cases)

Group	Count	Mean (SD)	Range
Canonical *NTRK* fusion	8	1.78 (1.57)	0.1 to 4.3
Noncanonical *NTRK* fusion	3	1.67 (0.5)	1.2 to 2.2
FUS *NTRK* fusion	9	−4.6 (1.74)	−6.2 to −1.1

**Figure 5:**
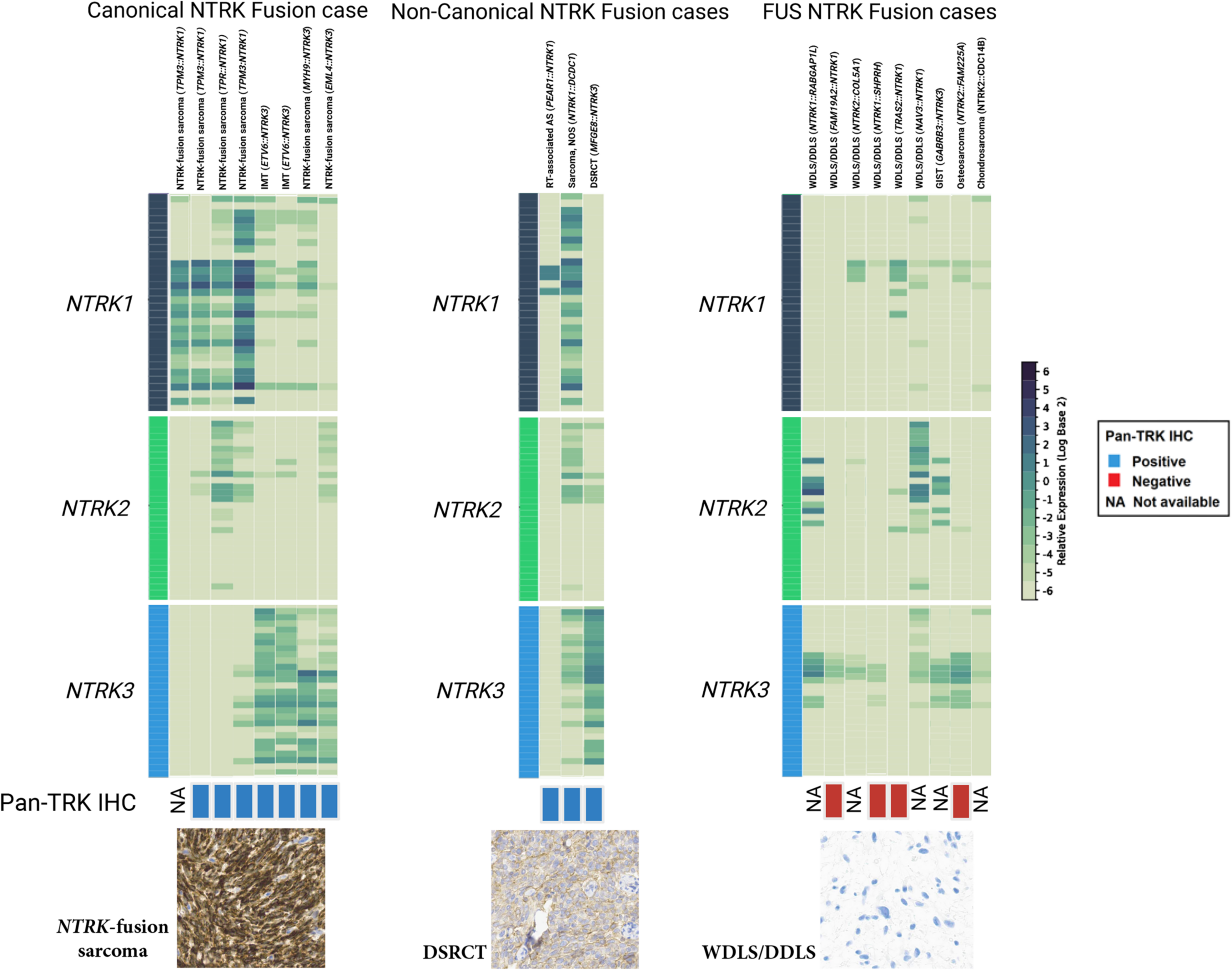
Relative *NTRK* RNA expression and Pan‐TRK immunohistochemistry correlation. Heatmap illustrating relative mRNA expression levels (Log Base 2) of *NTRK1, NTRK2*, and *NTRK3* across individual cases, grouped by fusion classification (canonical, noncanonical, and FUS). The color intensity corresponds to expression levels as indicated by the adjacent scale bar. Below the heatmap, the Pan‐TRK immunohistochemistry (IHC) status (positive, negative, or not available) is shown for each case, with representative IHC images provided for an *NTRK*‐fusion sarcoma (canonical), DSRCT (noncanonical), and WD/DDLS (FUS). DSRCT, desmoplastic small round cell tumor; GIST, gastrointestinal stromal tumor; IMT, inflammatory myofibroblastic tumor; NOS, not‐otherwise specified; RT‐associated AS, radiation‐associated angiosarcoma; WDLS/DDLS, well‐differentiated/dedifferentiated liposarcoma.

Moreover, Pan‐TRK immunohistochemical stain (whenever available) was positive in all cases with oncogenic *NTRK* fusions (seven in canonical group and three in noncanonical group), and negative in FUS cases (*n* = 4) (Figure 5).

A comparison of the two main cohorts reveals distinct clinicopathologic and genomic profiles (Table 1). The oncogenic fusion group is characterized by histotypes known to be driven by kinase fusions, a younger patient demographic, and a relatively simple genomic background, and higher mRNA *NTRK* expression, with the notable exception of *CDKN2A/B* deletions in a subset of *NTRK1*‐rearranged tumors. Conversely, the FUS group is defined by histologies with established alternative driver events (e.g., *MDM2* amplification in liposarcoma), an older patient demographic, and a more complex genomic landscape with a higher fraction of genome altered, and almost null expression of mRNA *NTRK*.

Of note, none of the 15 cases in the FUS/Indeterminate group were enrolled in *NTRK* inhibitors as part of their systemic treatment.

## Discussion

*NTRK* gene fusions have been identified as oncogenic drivers in a broad spectrum of adult and pediatric solid tumors. While the overall prevalence is low, estimated at approximately 0.3% of all solid malignancies, these fusions are highly enriched in several rare cancer types [12]. They are considered pathognomonic for entities such as infantile fibrosarcoma, cellular congenital mesoblastic nephroma, and secretory carcinoma of the breast and salivary gland, which are characterized by the *ETV6‐NTRK3* fusion [7, 13, 14]. The in‐depth understanding of the biology of these fusion‐driven tumors has led to a paradigm shift in cancer therapy. The development of highly potent and selective TRK inhibitors, such as larotrectinib and entrectinib, has demonstrated remarkable and durable clinical responses across diverse tumor types harboring *NTRK* fusions, marking these fusions as key therapeutic targets [15, 16].

Our study demonstrates a significant caveat to this paradigm within mesenchymal neoplasia; a substantial proportion (31%) of *NTRK* fusions detected by broad genomic profiling, particularly in sarcomas with complex karyotypes, do not appear to be the primary oncogenic drivers. These findings underscore the necessity of a multi‐faceted diagnostic approach that integrates molecular results with histopathologic context and stringent criteria for functionality to distinguish *bona fide* driver fusions from passenger events.

Brahmi *et al* [17] reported *NTRK* gene fusions in three cases of sarcoma subtypes characterized by concurrent *MDM2/CDK4* amplification (two dedifferentiated liposarcoma and one intimal sarcoma). Molecular analysis via RNAseq identified specific *NTRK* fusion transcripts in each case: *GON4L::NTRK1*, *VAMP4::NTRK1*, and *RAB14::NTRK3*, all demonstrating high levels of *NTRK* gene expression. Based on these findings, the authors concluded that sarcomas with *MDM2* and/or *CDK4* amplification, including both liposarcomas and intimal sarcomas, should be included in routine *NTRK* fusion testing. However, the functional significance of these *NTRK* fusions was not clinically demonstrated in any of the three patients, as no protein expression was documented, and none received a TRK‐fusion‐inhibiting drug. Our findings, however, present a contrasting perspective, particularly regarding the functional significance of *NTRK* fusions in the context of *MDM2/CDK4* amplified sarcomas. In our cohort, relative mRNA expression levels of *NTRK* genes (Log Base 2) showed that the FUS group demonstrated significantly lower expression, with a mean of −4.60 compared to the oncogenic group (*p* < 0.001). This marked downregulation in the FUS group, which notably includes cases with *MDM2/CDK4* amplification, is further supported by our immunohistochemical findings, Pan‐TRK, that was consistently negative in selected FUS cases, suggesting that these fusions are unlikely to be functionally active or driving oncogenesis in these tumors. This directly challenges the broad recommendation for routine *NTRK* fusion testing in *MDM2/CDK4* amplified sarcomas, as the mere presence of a fusion transcript, particularly at low expression levels, may not indicate clinical relevance or therapeutic susceptibility to TRK inhibitors. None of the 15 cases in our FUS/Indeterminate group were enrolled in *NTRK* inhibitor trials as part of their systemic treatment, aligning with our interpretation of their nondriver status.

Our cohort segregated into two distinct clinicopathologic and genomic groups. The first, which was classified as having oncogenic fusions, consisted largely of tumors now recognized as canonical *NTRK*‐driven entities, such as *NTRK*‐rearranged spindle cell neoplasm, lipofibromatosis‐like neural tumor, and infantile fibrosarcoma [12, 14, 18]. These neoplasms were characterized by a significantly younger median age at diagnosis and a relatively quiet genomic background, with low tumor mutational burden and fraction of genome altered. This is consistent with the established model of fusion‐driven sarcomas, where the fusion is the primary initiating event and the tumors are typically genomically stable, lacking other significant driver mutations [19, 20]. The almost exclusive association of *CDKN2A/B* homozygous deletions with oncogenic *NTRK1* fusions in our series is a notable finding, which has been corroborated by other recent studies [21, 22], reporting *CDKN2A* deletion in 33–55% of cases with *NTRK*‐driven spindle cell neoplasms, suggesting a potential cooperative role in tumorigenesis within this specific context. We also found that the presence of *CDKN2A/B* deletions was associated with aggressive clinical behavior, with four out of seven patients developing local recurrence or distant metastasis and three patients dying of disease.

Conversely, the group comprising fusions of uncertain significance (FUS) arose in tumors with complex, unstable genomes and well‐defined alternative drivers. The most striking example was the enrichment of *NTRK* fusions in well‐differentiated/dedifferentiated liposarcomas (WD/DDLPS), which constituted 59% of our FUS group. These tumors are defined by the pathognomonic amplification of the 12q13–15 region, including the *MDM2* and *CDK4* oncogenes, and are absent from the oncogenic *NTRK* group. Additionally, rare reports of intimal sarcomas, another histotype characterized by gene amplifications including *MDM2/CDK4*, harboring *NTRK* fusions have been reported [17]. However, in our cBioPortal cohort of 24 cases of intimal sarcoma with DNA NGS results (data not shown), none showed *NTRK* fusions. The co‐occurrence of *NTRK* fusions with *MDM2*/*CDK4* amplification has been recently documented [15, 23], challenging the prior consensus from the World Sarcoma Network that *MDM2*‐amplified sarcomas could be excluded from routine *NTRK* testing [24]. Our data, representing the largest series to date, strongly suggest that in the context of a dominant driver, such as *MDM2* amplification, *NTRK* fusions are likely passenger events related to the significant genomic instability that characterizes DDLPS. This is further supported by the higher fraction of genome altered (FGA) and a trend towards more frequent *TP53* mutations in our FUS group, hallmarks of genomic complexity [25].

The concept of passenger fusions in genomically complex sarcomas is not without precedent. Ameline *et al* [26] described *NTRK* rearrangements in three osteosarcomas, another sarcoma defined by a complex karyotype, all of which were nonfunctional and occurred in the setting of other genomic alterations, including *CDKN2A/B* deletions. Hence, the functional significance of chimeric transcripts in the context of genomically unbalanced tumors should be interpreted with caution, a conclusion our findings strongly reinforce. The critical determinant of a fusion's oncogenic potential lies in its structure: a canonical driver fusion must be expressed at the RNA level, remain in‐frame, and preserve the complete kinase domain of the 3’ *NTRK* partner, which is then constitutively activated by a dimerization domain from the 5′ partner [2, 27]. Our study rigorously applied these criteria, classifying fusions as FUS if they were out‐of‐frame, predicted a truncated kinase domain, or were not detected by RNA sequencing. The high rate of discordance between DNA‐based structural variant detection and RNA‐based confirmation in our cohort mirrors the findings of Solomon *et al*, who reported that 43% of *NTRK* structural variants detected by DNA sequencing did not result in a transcribed fusion [23]. This emphasizes that RNA‐based sequencing is the gold standard and an indispensable tool for validating the expression and structure of a potential fusion, especially in complex genomic settings where random chromosomal rearrangements are common.

Our findings enlighten the crucial difference between oncogenic *NTRK* fusions in *NTRK*‐defined entities and *NTRK* FUS seen in other sarcomas, challenging the need for refining *NTRK* fusion screening proposed by Vanacker *et al* [28]. In their study, *NTRK* fusions were detected in 57 of 3,015 samples (1.8%) in four distinct pathogenic groups: group 1 (biologically irrelevant fusions lacking functional kinase domains), group 2 (technically false positive fusions), group 3 (*NTRK*‐defined subtypes with canonical fusion partners), and group 4 (non‐*NTRK* defined subtypes with uncertain clinical significance). RNAseq‐based expression profiling revealed limited clustering organization, with samples from groups 1 and 2 particularly dispersed, while groups 3 and 4 showed closer alignment along dimension 1 of the UMAP analysis. The biological relevance of *NTRK* fusion partners differed substantially between groups: group 3 contained predominantly canonical partners previously reported in *NTRK*‐translocated cancers (96% of cases), whereas none of the three group 4 samples had previously reported fusion partners, raising questions about their biological significance. Notably, two of the three non *NTRK‐*rearranged mesenchymal tumors in group 4 harbored *MDM2* amplification (one dedifferentiated liposarcoma and one intimal sarcoma) and the clinical targetability of *NTRK* fusions in these *MDM2*‐amplified or genetically complex sarcomas remains undemonstrated.

Our study also highlights the different testing pathways that lead to the discovery of these two distinct fusion categories. The oncogenic fusions were predominantly identified through pathologist‐initiated RNA sequencing on tumors with a suggestive histomorphology (e.g., spindle cell neoplasms), reflecting a diagnostic search for a defining molecular event. Conversely, the FUS group was almost exclusively identified through clinician‐initiated DNA sequencing panels in patients with advanced, often refractory, sarcomas, representing a therapeutic search for any actionable target. While a confirmed oncogenic *NTRK* fusion predicts a high likelihood of response to TRK inhibitors, with reported objective response rates of 58–79% in sarcoma patients [29, 30], the therapeutic benefit in a patient whose tumor is primarily driven by *MDM2* amplification and harbors a passenger *NTRK* fusion is questionable and likely to be minimal. Clinical trial data on the efficacy of TRK inhibitors in this specific FUS population are currently lacking.

The clinical utility of identifying oncogenic *NTRK* fusions is further underscored by the emergence of acquired resistance mechanisms following TRK inhibitor therapy. In our oncogenic *NTRK* fusion group, four patients developed secondary *NTRK* single nucleotide variants in the kinase domain, all of which were sampled from recurrent or metastatic lesions after treatment with larotrectinib or entrectinib. These included the solvent‐front mutations *NTRK1* p.G595R and *NTRK3* p.G623R, as well as the gatekeeper mutation *NTRK3* p.F617I and the xDFG mutation *NTRK1* p.G667C (involving the residue located adjacent to the critical DFG motif in the kinase domain) [31]. One patient notably acquired sequential mutations (*NTRK1* p.G595R followed by p.G667C) in successive lung metastases. These specific substitutions are well‐documented on‐target resistance alterations that interfere with drug binding through steric hindrance or conformational changes [16, 31, 32, 33]. In contrast, none of the cases in our FUS/Indeterminate group received TRK inhibitors, further reflecting the clinical distinction between these two categories.

This study has limitations, including its retrospective nature and the heterogeneity of sequencing assays used for initial detection. However, by applying a uniform and stringent set of criteria for classification and leveraging a large, well‐annotated sarcoma cohort, we provide a clear framework for interpreting *NTRK* fusions in mesenchymal tumors.

In conclusion, our findings demonstrate that while canonical, oncogenic *NTRK* fusions define a specific subset of genomically stable sarcomas, a significant number of *NTRK* fusions detected in other sarcoma types, particularly in genomically complex tumors like DDLPS, represent nonfunctional passenger events. The critical evaluation of a fusion's structural integrity via RNA sequencing and its genomic context, especially the presence of other established primary drivers, is essential to guide appropriate patient selection for TRK‐targeted therapy.

## Author contributions statement

CRA provided the study concept, supervised the research, performed the final review, and edited the manuscript. MAY collected the study data, performed the clinicopathologic review, conducted the colorimetric and statistical analyses, and drafted the manuscript. PS contributed to the molecular methodology and Archer FusionPlex data analysis. CS and MH performed the clinicopathologic review and provided critical revisions to the manuscript. All authors approved the final version of the manuscript.

## Data Availability

The data that support the findings of this study are available from the corresponding author upon reasonable request.
